# Human papillomavirus serology and tobacco smoking in a community control group

**DOI:** 10.1186/s12879-014-0737-3

**Published:** 2015-01-09

**Authors:** Karl T Kelsey, Heather H Nelson, Stephanie Kim, Michael Pawlita, Scott M Langevin, Melissa Eliot, Dominique S Michaud, Michael McClean

**Affiliations:** Department of Epidemiology, Brown University School of Public Health, Providence, RI USA; Department of Pathology and Laboratory Medicine, Brown University School of Medicine, Providence, RI USA; Masonic Cancer Center, Division of Epidemiology and Community Health, University of Minnesota, Minneapolis, MN USA; Research Program Infection and Cancer, German Cancer Research Center, DKFZ, Heidelberg, Germany; Department of Environmental Health, University of Cincinnati School of Medicine, Cincinnati, OH USA; Department of Environmental Health, Boston University School of Public Health, Boston, MA USA

**Keywords:** HPV, Smoking, Serology

## Abstract

**Background:**

HPV infection is an established risk factor for oropharyngeal cancer, and it has been proposed that cigarette smoking may potentiate HPV infection in the oral epithelium. We sought to test the hypothesis that cigarette smoking increases HPV infection in an HPV16 serology study of cancer-free individuals.

**Methods:**

Subjects were participants in a risk factor study for head and neck cancer, and were required to have no prior history of either HNSCC or any other cancer. Tobacco use and other risk factor data were gathered through interviewer-assisted questionnaires, while serology was conducted in a blinded fashion using a glutathione S-transferase capture enzyme-linked immunosorbent assay (ELISA) to detect antibodies against HPV16 L1, E1, E2, E4, E6 and E7 proteins. The differences in tobacco use by HPV serology were evaluated by ANOVA; and the reported odds ratios and 95% confidence intervals were determined by using unconditional logistic regression.

**Results:**

We found no overall association of HPV16 serological markers with smoking. However, when the data were stratified by median age, smoking was positively associated with seropositivity for the HPV16 L1 capsid antigen in the younger controls while the older controls were less likely to be HPV16 L1 positive if they smoked (p_interaction_ < 0.002). There was no similar association of smoking and age with serological response to the early proteins (i.e E6, E7).

**Conclusions:**

Exposure to HPV16 capsid protein (L1) is increased among relatively younger adults who smoke and diminished among older smokers. However, this pattern is not accompanied by a differential susceptibility for active infection (as determined by the early gene proteins such as E6 and E7) among young and older smokers.

## Background

Human papillomavirus (HPV) is currently accepted as a risk factor for squamous cancers of the anogenital region and head and neck (HNSCC). HPV16 is estimated to account for the majority of cervical cancers and approximately 90% of all of virus-positive head and neck cancers [1], although other high-risk HPVs are known to cause both of these cancers [2]. HPVs show a distinct preference for site of infection in the upper aerodigestive tract, being most commonly associated with infection of the base of the tongue, tonsillar bed, and oropharynx. The majority of incident cancers at these sites diagnosed in the United States today are HPV-associated, and current trends suggest that the incidence of HPV16-positive HNSCC will increase markedly in the near term [3].

Recent studies suggest that the population prevalence of any oral HPV in the United States is approximately 6.9% [4], with the prevalence of high-risk HPV16 being much less, estimated at 1-3% [4]. It is thought that most HPV infections are transient and spontaneously resolve. However, in a subset of individuals, high-risk HPV infection can persist, increasing the risk of viral integration into the host DNA, which can ultimately lead to HNSCC. There is currently considerable interest in understanding the factors that contribute, either together or independently, to increase exposure and infection by HPV in the oral cavity and pharynx.

There remains some controversy about whether or not smoking is associated with enhancing HPV-positive HNSCC [5,6], or if these risk factors are independent [7]. A related but separate question is whether smoking promotes HPV infection via shared risk behaviors, or perhaps gives rise to an altered immune response to the virus, potentially leading to a longer duration of infection (poorer clearance of the virus). Gillison et al [4,8] have recently shown that PCR detection of HPV from oral rinse in a subset of the National Health and Nutrition Examination Survey (NHANES) participants was positively associated with the current number of cigarettes smoked. At the same time, a recent prospective study of college-age men, using a both a measure of HPV16 serology and a method that collected exfoliated genital cells for HPV DNA detection, reported a modest increase in seroconversion associated with smoking [9], consistent with some of the literature [10,11]. Similarly, Kreimer et al recently showed that smoking is associated with detectable oncogenic HPV in oral rinses from normal men, but the incidence of infection was low (1%) and 5 of the 6 incident infections cleared within 12 months [12]. Others, however, have reported conflicting results [13-15], finding little evidence for smoking to enhance HPV infection. Given the still conflicting findings, the association between smoking and infection remains unclear. As these prior studies have examined the numerous different means of assessing HPV, often using DNA-based measurements, we have taken a more focused approach to the question and used serologic endpoints to assess the smoking-HPV relationship.

We have examined HPV serological data in cancer-free controls enrolled in a population-based case-control study of HNSCC. Of interest to us was the nature of the association of evidence of an immune response to HPV with an individual’s history of tobacco use. We have examined both L1 (capsid) antibodies, which are formed after exposure to the virus and do not necessarily indicate recent infection, as well as antibodies to early gene products, which effectively signal relatively recent pathological infection, asking whether tobacco smoking is associated with the presence of these HPV biomarkers.

## Methods

### Study population

Incident cases of HNSCC were enrolled through nine medical facilities located in Boston, Massachusetts (Brigham and Women's Hospital, Beth Israel Deaconess Medical Center, Boston Veterans Administration, Boston Medical Center, Dana-Farber Cancer Institute, Harvard Vanguard Medical Associates, Massachusetts Eye and Ear Infirmary, Massachusetts General Hospital, and New England Medical Center) as part of a population-based case-control study of head and neck cancer in the greater-Boston area [7]. Control subjects with no prior history of either HNSCC or any other cancer were selected using town records and frequency-matched to cases on age (+/- 3 years), sex, and neighborhood/town of residence. The study includes data collected from two periods of recruitment from the same population: Phase I was conducted between December 1999 and December 2003 (685 controls and 533 cases) and Phase II was conducted between October 2006 and June 2011 (567 controls and 509 cases). All subjects enrolled in the study provided written informed consent as approved by the Institutional Review Boards of the nine medical facilities and of the Harvard School of Public Health. The participation rate for controls was 47%. A total of 1107 controls provided blood upon enrollment during the two phases.

### HPV16 serology measurement

Serum was separated from venous blood within 12-24 hr of blood drawing and stored at -80°C. To detect antibodies against HPV16 L1, E1, E2, E4, E6 and E7 proteins, a glutathione S-transferase capture enzyme-linked immunosorbent assay (ELISA) was used in combination with fluorescent bead [16,17]. This assay detects HPV antibodies with high type specificity and demonstrates assay sensitivity similar to the “gold-standard” for L1-serology that uses virus-like particles (VLP) as antigens [17].

### Additional exposure assessment

All study subjects responded to a self-administered questionnaire to collect data on demographic characteristics, medical history, family history of cancer (first-degree relatives), detailed smoking and drinking habits, occupational history, and residential history. Questionnaires were mailed to cancer-free control subjects and were returned and reviewed by study personnel during the in-person visit. Information on the demographic characteristics such as age, sex, race, education, and income, as well as smoking pack-years and average drinks per week, have been previously collected and defined [7].

### Statistical analysis

Differences in tobacco usage by HPV16 serological status, controlled for other risk factors and characteristics, were assessed using ANOVA. Odds ratios (OR) and 95% confidence intervals (CIs) were estimated for smoking status using unconditional logistic regression and controlling for known risk factors, including age, sex, race (white or other), average drinks per week (continuous), education (less/high school graduate or more), income, number of sexual partners and age at first intercourse. We created a variable for any positive serology for oncogenic proteins (E6/E7) and L1 for HPV type 16. All analyses were conducted in R (Version 2.14) and all tests were two-sided.

## Results

The demographics of the study population are shown in Table 1, stratified by their HPV16 serological status. Controls who described themselves as non-white and those whose self-reported annual income was below $50,000 were significantly more likely to have some positive serological measure of HPV16. Those who reported their age at first intercourse to be under 18 years old were similarly significantly more likely to have some detectable positive serological measure of HPV16. Interestingly, there was no association of number of sexual partners, education, age, sex or smoking or drinking history with HPV16 serology in the unadjusted data.

**Table 1:** **Descriptive statistics of subjects by HPV 16 serology status**

**Characteristic**	**All HPV16 negative (N = 767)**	**Any HPV 16 Pos (N = 339)**	P-value*
Age			
Mean (sd)	60.7 (10.7)	60.7 (11.7)	0.99
Sex, n (%)			
Male	567 (73.9)	248 (73.2)	
Female	200 (26.1)	91 (26.8)	0.82
Race, n (%)			
White	707 (92.2)	290 (85.5)	
Other	59 (7.7)	49 (14.5)	0.002
Education**, n (%)			
At least some college	545 (71.1)	229 (67.6)	
HS diploma or less	201 (26.2)	102 (30.1)	0.21
Income**^b^, n (%)			
< $50,000	242 (31.6)	129 (38.1)	
≥ $50,000	450 (58.7)	171 (50.4)	0.02
Smoking pack-years, n (%)			
None	311 (40.5)	131 (38.6)	
First tertile (>0 to 16)	149 (19.4)	69 (20.4)	
Second tertile (16 to 22)	156 (20.3)	69 (20.4)	
Third tertile (22+)	151 (19.7)	70 (20.6)	0.93
Average drinks per week, n (%)			
First quartile (<2.2)	193 (25.2)	82 (24.2)	
Second quartile (2.2-5.5)	192 (25.0)	84 (24.8)	
Third quartile (5.5-13.6)	198 (25.8)	78 (23.0)	
Fourth quartile (13.6+)	181 (23.6)	95 (28.0)	0.45
Age of intercourse^b^, n (%)			
<18 years	155 (20.2)	95 (28.0)	
≥18 years	295 (38.5)	118 (34.8)	0.01
Number of sexual partners^a,b^, n (%)			
<16	165 (46.5)	95 (45.9)	
16+	165 (46.5)	99 (47.8)	0.86

*Fisher’s exact test used to calculate p-value; **Values do not add to 100% due to missing data.

^a^Phase 2 only.

^b^These characteristics were dichotomized at their median values.

In an effort to further examine the relationship of cigarette smoking with HPV16 serology, we examined different serological measures of HPV16 by cumulative smoking history (ever vs. never) (Table 2) and adjusted for age, race, sex, education, and average drinks per week. As there were missing data for income, age of first intercourse, and number of sexual partners, the initial models did not include these variables (Table 2). This analysis similarly showed no significant association of cumulative smoking with any individual measure of HPV16 or with a combined variable indicating any serological response. Adjustment for age of first intercourse did not appreciably alter these results.

**Table 2:** **Logistic regression models of smoking (ever/never) on HPV 16 serology**

**HPV16 serology measure**	**Smoking** ^**b**^ **status**	**Number negative/positive**	**OR** ^**a**^ **(95% CI)**	**p-value**
L1	Never	407/30	Ref.	-
	Ever	617/43	1.04 (0.62, 1.73)	0.89
E1	Never	431/6	Ref.	
	Ever	653/8	0.92 (0.30, 2.80)	0.88
E2	Never	417/20	Ref.	
	Ever	639/22	0.74 (0.38, 1.41)	0.34
E4	Never	355/82	Ref.	
	Ever	546/115	0.95 (0.68, 1.31)	0.74
E6	Never	431/6	Ref.	-
	Ever	642/19	2.30 (0.88, 6.03)	0.09
E7	Never	421/16	Ref.	-
	Ever	626/35	1.23 (0.65, 2.32)	0.52
Any HPV16	Never	308/129	Ref.	-
	Ever	453/208	1.12 (0.85, 1.48)	0.42

^a^Adjusted for age, race, sex, education, and average drinks per week.

^b^Defined as “ever” for participants who smoked 100 cigarettes or more (or >0 smoking pack-years and “never” for those who had not (or =0 smoking pack years).

Since some data have suggested that current smoking may be associated with HPV16 presence, we repeated this analysis (Table 3), stratifying the smokers by current, former and never smoking. In this analysis there was no clear significant association of current smoking with any individual or combined measure of positive HPV16 serology, although for E6 antibodies there was a borderline association between former-smoking and positive E6 serology.

**Table 3:** **HPV16 serology stratified by smoking status**

**HPV16 serology measure**	**Smoking** ^**b**^ **status**	**Number negative/positive**	**OR** ^**a**^ **(95% CI)**	**p-value**
L1	Never	407/30	Ref.	-
	Former	472/34	1.11 (0.65, 1.90)	0.70
	Current	130/9	0.92 (0.41, 2.07)	0.85
E1	Never	431/6	Ref.	
	Former	501/6	0.87 (0.26, 2.87)	0.82
	Current	137/2	1.25 (0.23, 6.68)	0.80
E2	Never	417/20	Ref.	
	Former	490/17	0.73 (0.37, 1.46)	0.38
	Current	135/4	0.64 (0.21, 1.98)	0.44
E4	Never	355/82	Ref.	
	Former	415/92	1.01 (0.71, 1.42)	0.97
	Current	117/22	0.82 (0.48, 1.40)	0.46
E6	Never	431/6	Ref.	-
	Former	491/16	2.53 (0.95, 6.81)	0.06
	Current	136/3	1.72 (0.40, 7.31)	0.46
E7	Never	421/16	Ref.	-
	Former	478/29	1.35 (0.70, 2.61)	0.36
	Current	133/6	0.94 (0.35, 2.54)	0.90
Any HPV 16	Never	308/129	Ref.	-
	Former	339/168	1.23 (0.92, 1.65)	0.16
	Current	101/38	0.86 (0.55, 1.34)	0.50

^a^Adjusted for age, race, sex, education, and average drinks per week.

^b^Smoking status defined by smoking pack-years. Participants who reported zero smoking pack-years were considered to have never smoked. Participants who reported >0 smoking pack years were divided into current and former status based on whether they reported to be current smokers upon entry into the study.

Finally, in an effort to examine the possibility that age was acting as an effect modifier, we repeated the analysis of the association of cumulative smoking with serological measures of HPV16, stratifying by the median age of the control population. For those under the median age of 61, smoking increased the risk of having antibodies to the L1 capsid protein, while at the same time decreasing the risk of being L1 positive in those over the age of 61 (p_interaction_ < 0.002; Table 4). Adjusting for age at first intercourse did not appreciably alter these findings. In contrast, no similar trend (by age) was observed for smoking associated variation in early gene protein antibody production (Table 4), although the point estimates for an association for E1 antibodies were similar (albeit not statistically significant) to those observed for L1 seropositivity.

**Table 4:** **HPV serology by ever vs. never smoked, stratified by age (above or below the median of 61 years)**

**HPV16 serology measure**	**Smoking status**	**Number negative/positive (<61 years)**	**OR** ^**a**^ **(95% CI) (<61 years)**	**p-value (<61 years)**	**Number negative/positive (≥61 years)**	**OR** ^**a**^ **(95% CI) (≥61 years)**	**p-value (≥61 years)**
L1	Never	241/11	Ref.	-	166/19	Ref.	-
	Ever	264/29	3.05 (1.42, 6.56)	0.004	353/14	0.31 (0.15, 0.66)	0.003
E1	Never	251/1	Ref.	-	180/5	Ref.	-
	Ever	288/5	4.38 (0.46, 42.1)	0.20	365/3	0.29 (0.07, 1.31)	0.11
E2	Never	244/8	Ref.	-	173/12	Ref.	-
	Ever	285/8	0.85 (0.29, 2.48)	0.77	354/15	0.65 (0.29, 1.48)	0.30
E4	Never	204/48	Ref.	-	151/34	Ref	-
	Ever	240/53	0.94 (0.59, 1.50)	0.80	306/62	0.95 (0.59, 1.53)	0.83
E6	Never	248/4	Ref.	-	183/2	Ref.	-
	Ever	283/10	2.44 (0.70, 8.43)	0.16	359/9	2.85 (0.58, 13.9)	0.19
E7	Never	243/9	Ref.	-	178/7	Ref.	-
	Ever	283/10	1.00 (0.37, 2.71)	0.99	343/25	1.50 (0.62, 3.64)	0.37
Any HPV 16	Never	179/73	Ref.	-	129/56	Ref.	-
	Ever	199/94	1.26 (0.85, 1.87)	0.26	254/114	1.00 (0.67, 1.48)	0.98

^a^Adjusted for age, race, sex, education, and average drinks per week.

## Discussion

In our cancer-free control population, income, self-reported race and age at first intercourse were associated with a significantly increased likelihood of having some measure of positive HPV16. This is consistent with a now large literature –(reviewed in [1,18]). Unlike many studies, we did not observe a significant association of number of sexual partners with HPV16 positivity, although there was a small increase in the estimate of risk in our data (Table 1).

Both the unadjusted and adjusted data showed no overall association of smoking with any serologic measure of HPV16 when considering participants of all ages (range: 22-91 years). However, stratifying by median age (61 years) revealed an increased risk of antibodies for HPV16 L1 associated with cigarette smoking in younger participants and a concomitant decreased risk for the same measure of HPV16 (antibodies to the L1 capsid protein) in older smokers. The observed increase in likelihood of detecting HPV16 L1 antibodies in the younger age group is supported by the findings of a recent population-based cross-sectional study of NHANES data, in which the authors found that self-report and biomarkers of recent smoking was associated with increased detection of HPV16 DNA in oral rinse samples among 18-59 year olds [8]. Age did not modify any other estimate of risk for positive serological response to HPV16; in particular, there was minimal indication of any modification of risk of detection of antibodies to any of the early gene proteins.

The interpretation of positive HPV16 serology can be complex. The detection of antibodies to the capsid proteins is most often thought to reflect exposure to the virus, while antibodies to the early genes reflect an active infection, since production of the early gene proteins requires virus-driven cellular mitosis. Hence, the prevalence of positive HPV16 L1 serology is considerably higher than that of the early genes (in our data and in that reported by others) [19-21]. In addition (and consistent with this interpretation of these biomarkers), many studies have shown that HNSCC risk is considerably greater for those with evidence of antibodies to early genes (particularly E6 and E7) [2,22]. In this context, our data suggests that cigarette smoking may modify either the exposure to the HPV16 virus, the immune response to exposure, or both of these, while not affecting the active formation of infections.

One explanation of our findings could be that the younger smokers were exposed to HPV16 quite often, while older smokers were less exposed to the virus. This is possible, of course, and it has been reported that smoking is associated with a enhanced risk-taking behavior [23]. We attempted to control for this by adjusting for age at first intercourse and number of sexual partners in our models. There may be residual confounding that is responsible for the finding of age-associated differences in HPV16 L1 antibodies modified by smoking.

It also is possible that smoking produces microtrauma, increasing opportunities for entry of the virus. However, for this to be consistent with our data, smoking induced microtrauma would need to be age-associated, and be worse in younger smokers. This is unlikely, since smoking is associated with significant dental pathology and this is known to worsen with age and continued smoking, increasing, rather than decreasing, opportunities for viral entry.

Alternatively, smoking-related variation in the ability of an individual to produce antibodies after HPV16 exposure (an enhanced response to HPV associated with smoking in younger people, with waning immunity in older smokers after prior exposure) could explain our findings. It is known that smoking alters leukocyte profile [24] and smokers have been shown to respond to vaccination with production of both more and less antibody under differing antigenic challenge [25,26]. If HPV16 exposure is relatively stable across the population, changes in the immune response to HPV16 that are associated with smoking (enhanced antibody production in younger smokers and less antibody response in older smokers) could produce our results. Of course, an altered immune response could also lead to less viral clearance, which would likely enhance the oncogenic effects of the virus. We cannot differentiate between these possibilities, although our data would suggest that tobacco smoking does alter either exposure or immune response (or both) to HPV16 differentially by age.

It is important to also note that our data provide no clear indication that active HPV16 infection (as represented by an immune response to the early gene proteins) is altered by smoking, at any age. At the same time, our study is limited by the small numbers of disease-free participants who were seropositive for the early gene proteins. While our findings for antibodies to the capsid proteins are robust, it is less clear that the findings for antibodies to the early genes are stable. Hence, while our data suggest that smoking does not enhance active infection, we believe that it is important to revisit the question of the relationship of tobacco use with HPV16 early gene positivity in large numbers of disease free individuals, as the rate of clearance of the virus may be affected by smoking.

## Conclusions

In summary, our data suggest that HPV16 exposure is modified by age and smoking. It is not clear whether the association of HPV16 capsid antigen positivity with smoking is also accompanied by an alteration in the pathological process of infection. It is becoming crucially important to assess whether smoking is able to impact cellular response to viral infection and the progress toward virally driven disease.
